# Distribution, genetic analysis and conservation priorities for rare Texas freshwater molluscs in the genera *Fusconaia* and *Pleurobema* (Bivalvia: Unionidae)

**DOI:** 10.1186/2046-9063-8-12

**Published:** 2012-06-25

**Authors:** Lyubov E Burlakova, David Campbell, Alexander Y Karatayev, Don Barclay

**Affiliations:** 1 Great Lakes Center, Buffalo State College, 1300 Elmwood Ave, Buffalo, NY, 14222, USA; 2 The Research Foundation of The State University of New York, Buffalo State College, Office of Sponsored Programs, Buffalo, NY, 14222, USA; 3 The Paleontological Research Institution, 1259 Trumansburg Road, Ithaca, NY, 14850, USA; 4 7219 FM 2781, Kennard, TX, 75847, USA

**Keywords:** Freshwater molluscs, *Fusconaia askewi*, *Fusconaia lananensis*, *Pleurobema riddellii*, Molecular identification, Taxonomy, Distribution, Habitat requirements, Conservation priorities

## Abstract

**Background:**

Freshwater bivalves in the order Unionoida are considered to be one of the most endangered groups of animals in North America. In Texas, where over 60% of unionids are rare or very rare, 15 species have been recently added to the state’s list of threatened species, and 11 are under consideration for federal listing. Due to insufficient survey efforts in the past decades, however, primary data on current distribution and habitat requirement for most of these rare species are lacking, thus challenging their protection and management. Taxonomic identification of endemic species based on shell morphology is challenging and complicates conservation efforts. In this paper we present historic and current distributional data for three rare Texas species, *Fusconaia askewi, F. lananensis,* and *Pleurobema riddellii*, collected during our 2003–2011 state-wide surveys and suggest appropriate conservation measures. In addition, we tested the genetic affinities of *Fusconaia* and similar species collected from eastern Texas and western Louisiana using *cox1* and *nad1* sequences.

**Results:**

We found that *F. askewi* still inhabits four river basins in eastern and northeastern Texas and can be locally abundant, while *P. riddellii* was found only in one river basin. *Pleurobema riddellii* was well-separated from *F. askewi* and grouped with the *P. sintoxia* clade. The sequences for *F. lananensis* were very similar to those for *F. askewi*, with a maximum difference of just over 1% for *nad1* and only 0.7% for *cox1*, similar to the variation between *F. askewi* alleles. Except for one low difference (1.55%) with the partial *cox1* sequence for *F. burkei*, all other *Fusconaia* populations, including those from the Calcasieu drainage, differed by over 2.3% for both genes.

**Conclusions:**

Our study suggested that *F. lananensis* is not a valid species, and it is likely that only one *Fusconaia* species (*F. askewi* or its probable senior synonym *F. chunii*) is currently present in East Texas, thus simplifying conservation efforts. Distribution range of both these regional endemics (*F. askewi* and *P. riddellii*) has been reduced in the last 80 years.

## Background

Molluscs are among the most threatened groups of animals on the planet [1], and freshwater bivalves in the order Unionoida are considered to be one of the most endangered groups of animals in North America [2-4]. Our long-term state-wide study of Texas mussels revealed that 65% of all Texas unionid species are rare, including all state and regional endemics, and most endemic species are very rare [5]. Being one of the top states in species diversity and endemism, Texas ranks fourth in terms of the number of species extinctions [6]. Damming, pollution, water extraction, and urban development have all negatively affected the freshwaters of Texas [7]. Fifteen rare freshwater mussel species were recently added to the state’s list of threatened species [8], and 11 of those are currently under consideration for federal listing by the U. S. Fish and Wildlife Service [9,10].

Biodiversity is a fundamental component of evolutionary potential, and species are the primary targets of the U.S. Endangered Species Act. Conservation laws and methods cannot be implemented until the endangered organism is properly clarified and its geographical range is known [11,12]. In particular, some of these rare species, *Fusconaia flava* (Rafinesque)*, F. askewi* (Marsh)*,* and *F. lananensis* Frierson, are currently reported from several drainages west of the Mississippi [13-15], but identifying specimens using shell morphology is challenging. Morphological variation in *Fusconaia* in the lower Mississippi drainage is especially complex [16]. Burdick and White [17] reported an unusual genetic type in *Fusconaia* from the northern and western Ozark region, which could represent a northern extension of *F. askewi*. *Pleurobema riddellii* (Lea) can also be very similar in shell features to *F. askewi*[16]. Johnson [18] synonymized *F. askewi* with *F. flava* (under the name *F. undata*).

In light of the difficulties, we used genetic data as an additional line of evidence. We sampled *Fusconaia* and similar species from river systems in eastern Texas and western Louisiana to test the genetic affinities of the species, using *cox1* and *nad1* sequences. In this paper we describe the geographical distribution and habitat requirements of rare *Fusconaia* spp*.* and *P. riddellii* and results of molecular genetic analyses to define their biogeography, proper taxonomic status, and suggest appropriate conservation measures.

## Methods

### Field surveys

In this manuscript we use results of our state-wide survey of unionids in Texas, USA (latitudes 33°50′ - 26°56′, longitudes 102°08′ - 93°31′) from 2003 to 2011 [5,19]. Mussels were surveyed at 463 sub-sites that were pooled into141 major sites, distributed among 66 waterbodies belonging to 11 major drainages in Texas. The study was carried out with an appropriate Scientific Research Permit issued by the Texas Parks and Wildlife Department (TPWD), and landowner permission for wildlife research was acquired from each property owner before entering their property, if the land was privately owned. Abiotic parameters (physical and chemical) were recorded at the sites using a HACH Hydrolab Quanta, measured parameters included: temperature (°C), pH, total dissolved solids (g/L), conductivity (μS/cm), and turbidity (ed. NTU). In addition, we recorded depth and the dominant substrate type using the following classification by particle size: bedrock; large boulders (>45 cm); boulders (>25 - 45 cm); cobble (>6 - 25 cm); gravel (>6 - 60 mm); sand (0.06 - 6 mm); mud/silt (<0.06 mm). Substrates in sampled East Texas sites were represented by sand (32%), sand and gravel (21%), silt (15%), clay (6%), and combinations of these. Unionid sampling was conducted via hand collection of both live and dead mussels, by wading in shallow water and by snorkeling. Due to poor water visibility, tactile searches (running fingers over the sediment, usually up to 15 cm deep, depending on substrate type) were used at all sites. Timed searches were used to detect the presence of mussels and species diversity [20,21] at each site, and if mussel assemblages were present, quantitative methods (from 5 to 28 randomly placed 0.25 m^2^ quadrats at a site, in average 9 quadrats covering area of 3.75 m^2^), or area -constrained searches (area searched were from 4 to 66 m^2^) were used for assessments of density [22,23]. Relative species abundance was calculated as a percentage of live specimens belong to this species collected at a site from the total number of all live mussels found at the same site, and used as an indicator of the species’ dominance in mussel assemblages. Collected mussels were identified based on shell morphology, counted, measured with calipers to the nearest mm, and then carefully rebedded into the sediment from which they were taken. Ten specimens of *Fusconaia* sp. from the Neches drainage and 5 from the Sabine drainage were sequenced for *cox1*. Five *Fusconaia* specimens from the Neches drainage (including one not amplified for *cox1*) and 3 from the Sabine drainage were sequenced for *nad1*. Two specimens of *P. riddellii* from the Neches drainage were sequenced for *cox1,* with one of them also sequenced for *nad1*. Voucher specimens were deposited in the Great Lakes Center (Buffalo State College) Invertebrate Collection, in the North Carolina State Museum of Natural Sciences (Raleigh, NC), and in the Invertebrate Zoology Collection of the National Museum of Natural History (Smithsonian Institution, Washington, D.C.). All *Fusconaia* species identified during our study (*F. askewi* and *F. lananensis*) and historical data reported from East Texas (*F. askewii*[24,25], *F. askewi*[15,26-30], *F. flava*[15]*, F. lananensis*[31-33]*, Quadrula askewi*[34,35], *Q. askewii*[25], *Q. chunii*[25,35], *Q. flava nasuta*[34], *Q. lananensis*[25,34,36]*, Q. undata chunii*[34], *Unio askewii*[24], *U. cerinus*[24,37]*, U. chunii*[24,37,38], were considered to be *F. askewi.* For justification see sections “Genetic analysis” in Results and Discussion.

### Genetic analysis

Specimens were preserved in ethanol in the field. DNA extraction used Qiagen DNA extraction kits. Portions of the *cox1* and *nad1* genes were amplified. Primers for *cox1* were 5'–GTTCCACAAATCATAAGGATATTGG–3' and 5'–TACACCTCAGGGTGACCAAAAAACCA–3', adapted from Folmer *et al.*[39] and primers for *nadh1* were 5'–TGGCAGAAAAGTGCATCAGATTTAAGC–3' and 5'–GCTATTAGTAGGTCGTATCG–3' [40,41]. The primer LoGlyR (5’–CCTGCTTGGAAGGCAAGTGTACT–3′) [42] served as an alternate reverse primer for *nadh1*. The forward primer UNIOCOII.2 from Walker *et al.*[43] and/or the reverse primer HCOout (CCAGGTAAAATTAAAATATAAACTTC [44]) provided good amplification for *cox1* for some species. PCR cycles were: 92°C 2 min; 92°C 40 sec 40°C 40 sec 72°C 90 sec 5x; 92°C 40 sec 50°C 40 sec 72°C 90 sec 25x; 72°C 10 min; hold 4°C. PCR products were purified using Qiagen QIAquick PCR purification kits and, if necessary, Qiagen gel extraction kits. Cycle sequencing used ABI Big Dye Terminator kits with thermal cycle parameters of 1°C per second ramp speed, starting with 1 min at 96°C followed by 26 cycles of 96°C for 10 sec, 49°C for 5 sec, and 60°C for 4 min, then 10 min at 60°C and hold at 4°C. The cycle sequencing products were purified with Qiagen DyeEx kits and then run on an automated sequencer.

The results for each strand were compared and aligned using BioEdit [45]. We analyzed the sequences, along with previously published sequences for other representatives of Pleurobemini with TNT [46]. An Additional file 1 contains sequences used for genetic analysis [see Additional file 1. Maximum parsimony analyses used 500 random replicates, using all the “new technology” methods (sectorial searching, ratchet, drift, and tree fusing), which greatly speed up the process of finding optimal trees over older approaches [46]. Jackknife analyses used 500 replicates, each using a random “new technology” parsimony search of 10 replicates.

## Results

### Genetic analysis

**Figure 1:**
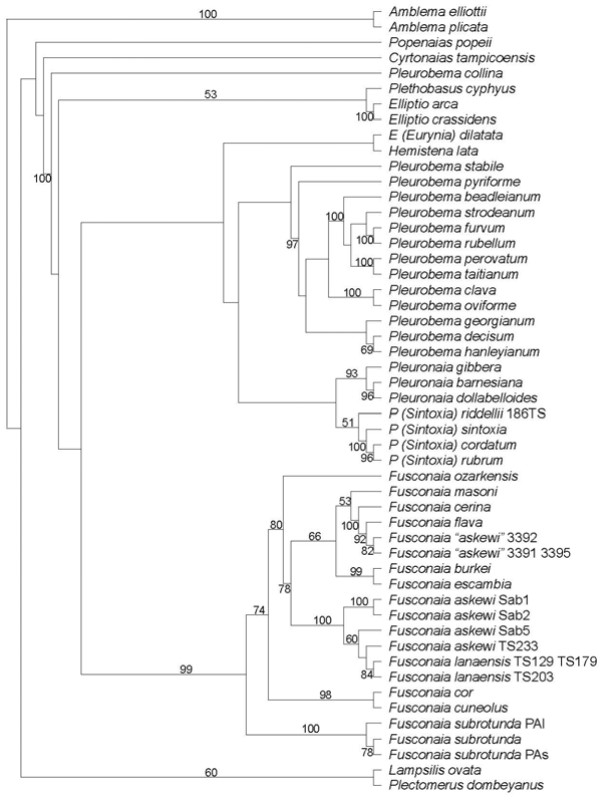
**Strict consensus cladogram, combined*cox1*and*nad1*data, with jackknife percentages shown if over 50%.** See text for discussion.

**Figure 2:**
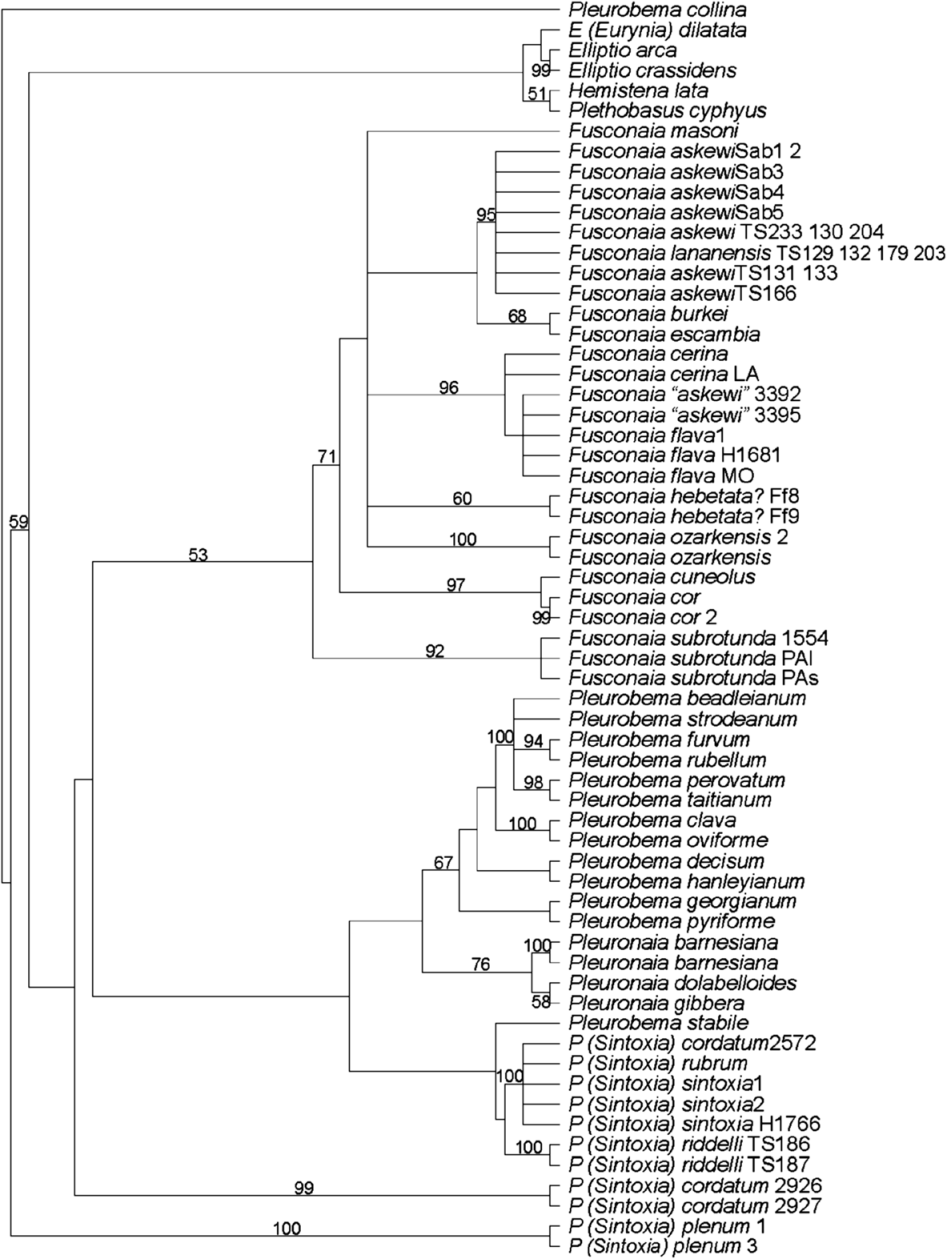
**Strict consensus cladogram,*cox1*data, with jackknife percentages shown if over 50%**.

**Figure 3:**
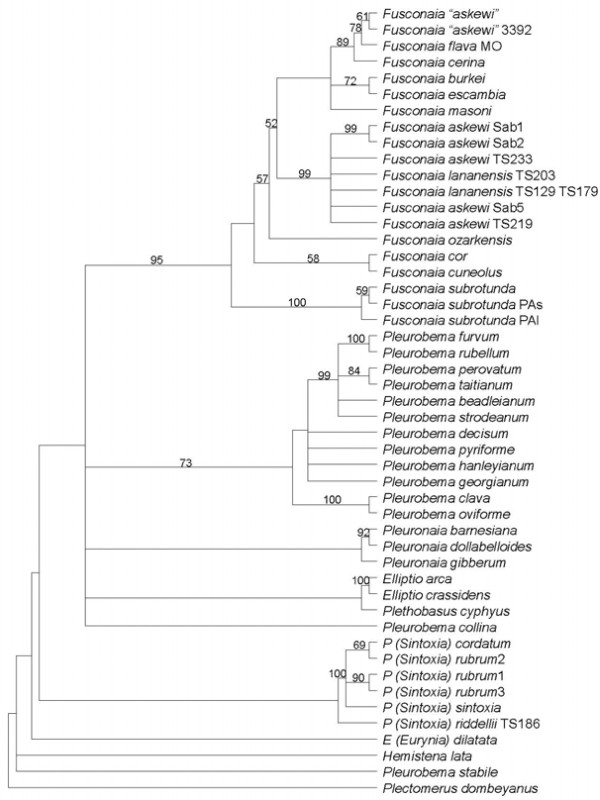
**Strict consensus cladogram,*nad1*data, with jackknife percentages shown if over 50%**.

The sequences for *F. lananensis* were very similar to those for *F. askewi*, with less than 1% difference, similar to the variation between *F. askewi* alleles (Tables 1,2). However, the sequences for *F. askewi* from the Sabine and Neches drainages differed from all other *Fusconaia* species by over 2.3% for both genes, except for the partial *cox1* sequence for *F. burkei*. In particular, the *cox1* sequences differed by no more than 0.7% between *F. askewi* and *F. lananensis*, typical of within-species variation, but differed by a minimum of over 2.5% from all other *Fusconaia* sequences, except the short sequence for *F. burkei*, fairly normal for species-level differences. The *cox1* sequences from putative *F. askewi* from the Calcasieu River system in Louisiana [47] differed from sequences for *F. flava* and *F. cerina* by less than 2% and in most cases by less than 1% (Table 1). One published sequence for *F. flava* (AF231733, [48] was identical to one of the Calcasieu sequences. Figures 1,2 and 3 show the phylogenetic analyses. Jackknife percentages close to 100 show strong support for a particular group. As cladograms, their branching sequence provides the important information. Thus, in Figure 1*Pleurobema (Sintoxia) riddellii* 186TS is modestly supported (51%) as being most closely related to the strongly supported (100%) group including *P. (Sintoxia) sintoxiaP. (Sintoxia) cordatum*, and *P. (Sintoxia) rubrum*. Those four in turn are most closely related to the group of the three *Pleuronaia* species. However, this association of *Pleuronaia* and *P. (Sintoxia)* received less than 50% jackknife support and was not supported by all of the analyses. The two *Fusconaia lananensis* have good support (84%) as being each other’s closest relative, and there is very strong support (100%) for a group including the Sabine and Neches *F. askewi* as well as *F. lananensis*. In turn, this *F. askewi-lananensis* group has fairly good support (78%) as being most closely related to the group including *F. masoni, F. cerina, F. flava*, the putative *F. askewi* from the Calcasieu, *F. burkei*, and *F. escambia*. The Calcasieu *Fusconaia* specimens are strongly supported (92%) as being most closely related to *F. flava*. In Figure 2*P. riddelli* again appears to be most closely related to *P. rubrum, P. sintoxia*, and *P. cordatum* 2572, but yet again this result is not well-supported. Multiple branches coming from a single vertical line indicates that the relationship among those branches is unresolved. Figure 2 shows strong support (95%) for a group including the Sabine and Neches *F. askewi* and the *F. lananensis* specimens, but does not tell anything about relationships among those eight sequences. Relationships among the different groups within *Fusconaia* are not well-resolved in Figure 2. Similarly, Figure 3 has strong support (99%) for a group of all of the *F. lananensis* and Sabine and Neches *F. askewi*, but apart from strong support (99%) for a group of *F. askewi* Sab1 and Sab2, does not support any particular relationships within that group. Again, *P. riddellii* receives weak support as being most closely related to *P. sintoxia, P. rubrum*, and *P. cordatum*.

**Table 1:** **Percent differences in*cox1*sequence for*Fusconaia*species**

	***F. askewi* 3392**	***F. askewi* 3395**	***F. askewi* Sab1 2**	***F. askewi* Sab3**	***F. askewi* Sab4**	***F. askewi* Sab5**	***F. askewi* TS131 133**	***F. askewi* TS166**	***F. askewi* TS233 130 204**	
*F. askewi* 3395	0.16									
*F. askewi* Sab1 2	3.94	4.12								
*F. askewi* Sab3	4.23	4.41	0.36							
*F. askewi* Sab4	4.48	4.68	0.57	0.19						
*F. askewi* Sab5	4.03	4.23	0.59	0.20	0.39					
*F. askewi* TS131, 133	4.08	4.24	0.35	0.54	0.57	0.59				
*F. askewi* TS166	2.72	2.64	0.53	0.55	0.60	0.32	0.43			
*F. askewi* TS233 130 204	3.73	3.91	0.35	0.18	0.19	0.20	0.30	0.22		
*F. burkei*	2.47	2.69	2.51	3.07	3.05	3.48	2.93	1.55	2.70	
*F. cerina*	1.16	1.54	4.49	4.80	5.09	4.65	4.59	3.57	4.26	
*F. cerina* LA	0.66	0.92	3.76	4.04	4.29	3.83	3.76	2.87	3.44	
*F. cor*	4.77	4.65	4.88	5.20	5.53	5.53	5.03	4.05	4.85	
*F. cor* 2606	4.60	4.55	4.71	5.02	5.34	5.33	4.92	3.97	4.75	
*F. cuneolus*	4.26	4.24	3.60	3.88	3.91	3.85	3.94	2.65	3.62	
*F. escambia*	10.37	10.63	10.03	10.63	10.61	10.84	10.40	7.39	10.40	
*F. flava* H1681	0.16	0.47	3.76	4.04	4.28	3.82	3.73	2.55	3.40	
*F. flava* MO	0.33	0.61	3.94	4.23	4.48	4.03	3.92	2.86	3.59	
*F. flava* 1	0.66	0.62	4.14	4.62	4.91	4.46	4.13	2.92	3.97	
*F. hebetata?* Ff8	3.73	4.14	3.32	3.42	3.68	3.07	3.39	3.73	3.00	
*F. hebetata?* Ff9	3.09	3.56	3.56	3.90	4.20	3.87	3.59	3.99	3.20	
*F. lananensis* TS129 132 179 203	3.73	3.91	0.70	0.54	0.57	0.59	0.61	0.43	0.30	
*F. masoni*	2.51	2.78	3.58	3.48	3.69	3.62	3.44	2.87	3.12	
*F. ozarkensis*	4.24	4.22	4.32	4.62	4.90	4.87	4.41	3.79	4.08	
*F. ozarkensis* 3501	4.76	4.70	4.87	5.18	5.50	5.50	4.89	4.02	4.57	
*F. subrotunda* 1554	4.25	4.39	4.52	4.82	5.11	4.67	4.42	3.56	4.42	
*F. subrotunda* PA l	4.07	4.56	4.33	4.62	4.91	4.67	4.59	3.79	4.59	
*F. subrotunda* PA s	4.77	4.87	4.88	4.80	5.09	4.87	4.41	3.55	4.40	
	*F. burkei*	*F. cerina*	*F. cerina* LA	*F. cor*	*F. cor* 2606	*F. cuneolus*	*F. escambia*	*F. flava* H1681	*F. flava* MO	*F. flava* 1
*F. cerina*	3.15									
*F. cerina* LA	2.69	1.24								
*F. cor*	4.36	4.83	4.65							
*F. cor* 2606	4.36	4.59	4.39	0.17						
*F. cuneolus*	4.11	4.27	4.08	2.55	2.25					
*F. escambia*	8.61	11.68	10.63	11.53	11.53	11.23				
*F. flava* H1681	2.24	0.95	0.48	4.47	4.22	3.91	10.13			
*F. flava* MO	2.24	1.23	0.61	4.65	4.39	4.08	10.13	0.16		
*F. flava* 1	2.69	1.56	0.93	4.82	4.44	4.14	10.11	0.48	0.62	
*F. hebetata* Ff8	2.99	3.41	3.76	5.09	5.16	4.55	9.52	3.33	3.76	4.22
*F. hebetata* Ff9	2.38	2.82	3.18	4.43	4.54	4.15	8.84	2.73	3.18	3.62
*F. lananensis* TS129 132 179 203	2.93	4.26	3.44	4.85	4.74	3.61	10.67	3.40	3.59	3.97
*F. masoni*	2.24	3.12	2.47	4.65	4.55	4.40	9.89	2.24	2.47	2.82
*F. ozarkensis*	3.39	4.25	3.90	4.84	4.57	4.25	10.91	3.89	3.90	4.28
*F. ozarkensis* 3501	3.84	4.73	4.38	5.37	5.07	4.73	11.16	4.38	4.38	4.77
*F. subrotunda* 1554	3.64	4.59	4.24	3.95	4.06	4.08	10.13	3.90	4.23	4.46
*F. subrotunda* PA l	3.40	4.59	4.41	3.59	4.07	4.25	10.13	3.90	4.40	4.46
*F. subrotunda* PA s	4.10	5.07	4.72	3.95	4.06	4.24	10.91	4.39	4.71	4.95
	*F. hebetata* Ff8	*F. hebetata* Ff9	*F. lananensis* TS129 132 179 203	*F. masoni*	*F. ozarkensis*	*F. ozarkensis* 3501	*F. subrotunda* 1554	*F. subrotunda* PA l		
*F. hebetata* Ff9	1.30									
*F. lananensis* TS129 132 179 203	3.00	3.20								
*F. masoni*	2.99	2.41	3.43							
*F. ozarkensis*	4.15	3.57	4.40	3.58						
*F. ozarkensis* 3501	4.54	3.95	4.89	4.06	0.46					
*F. subrotunda* 1554	4.36	4.16	4.42	3.91	4.24	4.72				
*F. subrotunda* PA l	4.76	4.17	4.59	3.76	4.41	4.89	1.24			
*F. subrotunda* PA s	4.75	4.35	4.40	4.23	4.72	5.21	1.23	1.24		

**Table 2:** **Percent differences in*nad1*sequence for*Fusconaia*species**

	***F. askewi* 3391**	***F. askewi* 3392**	***F. askewi* Sab1**	***F. askewi* Sab2**	***F. askewi* Sab5**	***F. askewi* TS219**	***F. askewi* TS233**	***F. burkei***	***F. cerina***	
*F. askewi* 3392	0.24									
*F. askewi* Sab1	3.85	3.84								
*F. askewi* Sab2	3.80	3.79	0.26							
*F. askewi* Sab5	3.00	2.99	1.04	1.02						
*F. askewi* TS219	3.10	3.07	1.18	1.18	0.33					
*F. askewi* TS233	3.48	3.47	1.59	1.58	0.79	0.51				
*F. burkei*	2.39	2.39	3.34	3.10	2.58	2.51	3.19			
*F. cerina*	1.37	1.24	3.96	4.04	3.24	3.07	3.60	2.45		
*F. cor*	4.68	4.66	6.06	5.77	5.04	6.12	5.93	4.34	4.28	
*F. cuneolus*	4.51	4.49	6.23	6.12	5.57	6.11	6.29	4.01	4.62	
*F. escambia*	2.71	2.58	3.97	3.92	3.38	3.43	3.88	0.63	3.00	
*F. flava*	0.49	0.61	3.43	3.39	2.59	2.91	3.07	2.55	1.49	
*F. lananensis* TS129 TS179	2.71	2.69	0.91	0.90	0.13	0.17	0.66	2.71	3.12	
*F. lananensis* TS203	2.85	2.83	1.04	1.02	0.25	0.17	0.79	2.89	3.25	
*F. masoni*	2.55	2.54	4.17	4.17	3.34	3.24	3.92	2.32	2.81	
*F. ozarkensis*	4.38	4.34	5.50	5.15	4.61	5.19	4.86	4.53	4.69	
*F. subrotunda*	5.52	5.50	7.56	7.42	6.50	6.68	7.07	5.35	5.66	
*F. subrotunda* PA l	4.75	4.72	6.43	6.35	5.52	5.55	5.96	4.70	5.06	
*F. subrotunda* PA s	4.85	4.84	6.30	6.21	5.39	5.56	5.69	5.70	5.21	
	*F. cor*	*F. cuneolus*	*F. escambia*	*F. flava*	*F. lananensis* TS129 TS179	*F. lananensis* TS203	*F. masoni*	*F. ozarkensis*	*F. subrotunda*	*F. subrotunda* PA l
*F. cuneolus*	4.33									
*F. escambia*	4.50	4.17								
*F. flava*	4.50	4.66	2.82							
*F. lananensis* TS129 TS179	5.33	5.15	3.44	2.32						
*F. lananensis* TS203	5.54	5.36	3.59	2.46	0.12					
*F. masoni*	5.07	5.07	3.08	2.41	3.21	3.34				
*F. ozarkensis*	6.18	5.67	5.00	4.23	4.33	4.49	5.32			
*F. subrotunda*	6.17	5.16	5.51	5.67	6.17	6.39	6.21	7.04		
*F. subrotunda* PA l	6.19	5.18	5.24	4.85	5.21	5.36	5.71	6.68	1.26	
*F. subrotunda* PA s	6.21	5.68	5.62	4.97	5.08	5.21	5.85	6.57	1.30	1.11

### Distribution, densities, size structure, and habitat

#### Fusconaia askewi

**Figure 4:**
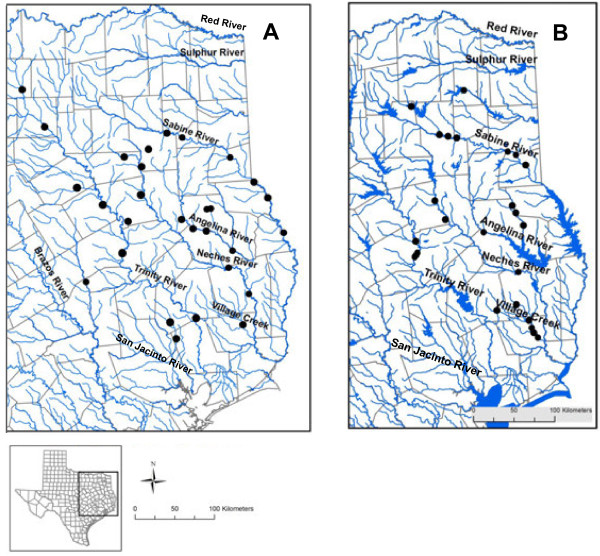
**Historical (before 1940, A) and current (1990-present, B) distribution of*Fusconaia askewi*in East Texas.** Historical data are from Pilsbry [38], Singley [37], Frierson [24], Frierson [36], Frierson [25], Strecker [34], and Bachtel [35]. Current data include authors’ data and literature records [15,26-33].

**Figure 5:**
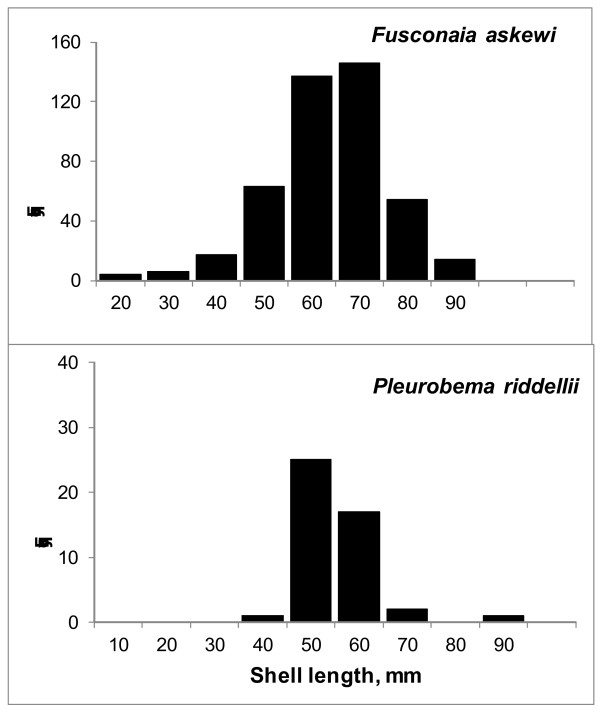
**Size-frequency distribution of live*Fusconaia askewi,*and*Pleurobema riddellii*shell length**.

A total of 931 live individuals was collected during our surveys (including 774 mussels originally identified as *F. askewi* and 157 identified as *F. lananensis*) at 25 sites in 17 East Texas counties (Anderson, Angelina, Cherokee, Hardin, Harrison, Houston, Jasper, Leon, Nacogdoches, Panola, Rusk, San Augustine, Shelby, Smith, Titus, Tyler, and Upshur) (Table 3, Figure 4B). We found *F. askewi* in four drainages (Neches, Trinity, Sabine, and Red river basins) in eastern and northeastern Texas. *Fusconaia askewi* was locally very abundant in Village Creek (Neches River basin), Neches, Sabine, Trinity and Angelina (Neches River basin) rivers, and in the Big Cypress Bayou (Red River basin). On average, *F. askewi* was the third most abundant species, and the number of live *F. askewi* collected at a particular site, on average, comprised 22% of the total number of all live mussels found at that site. Average density in mussel aggregations was 6.7 m^-2^ (Table 3). Sites with the greatest abundance were on Village Creek and the Neches and Sabine rivers. The most typical substrate for the species was sand, then a mixture of sand and silt, and gravel with sand. Average shell length of live *F. askewi* was 59.2 ± 0.6 mm (mean ± standard error here and elsewhere unless noted). Based on the presence of juveniles (Figure 5), the populations in East Texas were reproducing (shell length varied from 17 to 90 mm). Nevertheless we failed to find *F. askewi* in several waterbodies belong to the species’ former distribution range: in the San Jacinto River, its tributaries, and in Lake Houston, as well as in its historical location in Kickapoo Creek (North of Brownsboro, Henderson Co. [34] (Figure 4). Likewise, we did not find the species in any of the 6 reservoirs on the Trinity River and its tributaries. Our surveys also confirmed that *F. askewi* has been extirpated from Lanana and Bonita creeks (type localities for *F*. *lananensis*).

**Table 3:** **Historical and current distribution, and densities of*Fusconaia askewi*and*Pleurobema riddellii*in Texas**

**Habitat characteristics**	***F. askewi***	***P. riddellii***
Distribution (Literature data)	Angelina River, Attoyac Bayou, Bonita Creek, Lanana Creek, Cypress Bayou, Cypress River, Big Lake, Big Creek, Chambers Creek, Lake Fork Creek, Navasota River, Kickapoo Creek, Neches River, Sabine River, Sandy Creek, San Jacinto River, Trinity River, Village Creek and tributaries [14,15,24,26-29,31,34-36,49-53]	Angelina River, Big Lake, Kickapoo Creek, Sabine River, San Jacinto River, Trinity River, Village Creek and tributaries, Chambers Creek [15,24,30,31,34,35,37]
Current distribution (Our data)	Angelina River (27), Attoyac Bayou (25), Sandy Creek (52), Big Cypress Bayou (2), Neches River (274), Sabine River (129), Trinity River (36), Village Creek (386)	Angelina River (9), Neches River (86), Village Creek (37)
Density, m^-2^	6.7 ± 12.8 (data from 7 sites, 89 quadrats total)	1.9 ± 1.2 (5 sites, 49 quadrats)
Relative abundance, %	22 (1 – 58)	5 (1 – 13)

Amount of live molluscs found in each waterbody during this study is in parentheses. Densities in mussel assemblages (mean ± standard deviation) were calculated using 0.25 m^2^ quadrats. Relative species abundance (mean and range in parentheses) was calculated as a percentage of live specimens belong to this species collected at a particular site from the total number of all live mussels found at this site, and used as an indicator of the species’ dominance in mussel assemblages.

Only one dead shell and one valve of mussels identified as *F. flava* were found during our surveys, at two sites in the Sulphur River (Red River drainage), in Red River County and in Delta/Hopkins counties. Live individuals resembling *F. flava* have recently been collected in the East Fork of the Trinity River approximately 70 km from Dallas [54]. Mussels from the Sulphur River and the Trinity River have not been genetically tested yet.

#### Pleurobema riddellii

**Figure 6:**
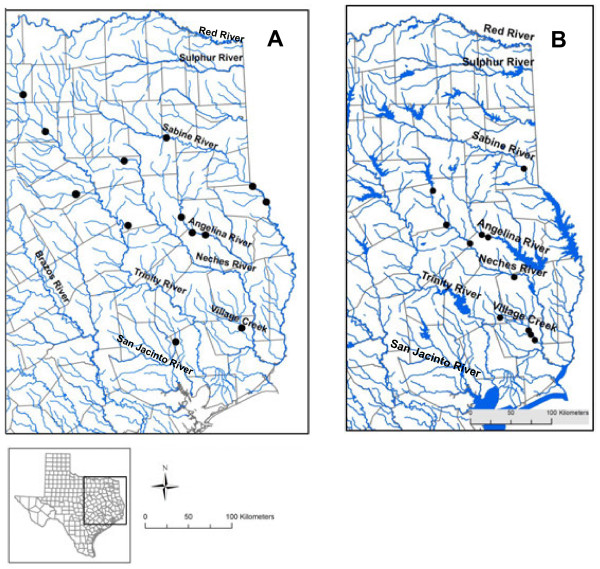
**Historical (before 1940, A) and current (1990-present, B) distribution of*Pleurobema riddellii*in Texas.** Historical data are from Frierson [24], Strecker [34], Bachtel [35]. Current data include authors’ data and literature records [15,30,31].

During our surveys, we found 132 live *P. riddellii* at 10 sites in 5 Texas counties (Anderson, Angelina, Cherokee, Hardin, and Nacogdoches), in the Neches, and Angelina rivers, and in Village Creek (Figure 6B, Table 3). Average density of *P. riddellii* was 1.9 m^-2^, and the species was not dominant in local unionid assemblages (the average relative abundance of *P. riddellii* was 5%, Table 3). Most often *P. riddellii* was found in sand, silty sand, and sometimes in a mixture of sand and clay. Mean and median *P. riddellii* length were 52.4 ±1.1 mm, range - 39–82 mm (Figure 5). The largest density was found in the Neches River south of Neches (Anderson Co.) in sand and gravel; this population had many juveniles (< 25 mm long) in 2009 (Barclay unpublished data).

#### Habitat requirements

We found that *F. askewi* and *P. riddellii* have similar distribution (Table 3) and very similar habitat requirements. All these species were found exclusively in lotic waters, in relatively shallow areas (at 0.2 - 1.5 m depth), and the most preferable substrates for both *F. askewi* and *P. riddellii* were sand, and combinations of sand with gravel and silt. Total dissolved solids among waterbodies studied varied from 0.10 to 0.15 g/L, turbidity – from 18.9 to 66.9 ed. NTU, pH – from 6.38 to 8.21. The lowest pH was recorded in Village Creek (average of 4 measurements in 2005 and 2007: 6.64 ± 0.24 (standard deviation), minimal 6.38 ± 0.12) and in Sandy Creek (6.69 ± 0.006). Minimal pH value for the studied rivers and creeks recorded from 1973 to 2009 was 4.8 (4.8 for Village Creek, 5.4 for the Angelina River, 5.6 for the Neches River, and 5.7 for Attoyac Bayou; data from the Texas Commission on Environmental Quality database (TCEQ Data Management and Analysis, Water Quality Planning Division), measured 4–12 times a year). This low pH caused heavy erosion of *F. askewi* shells, as it was previously recorded for *Corbicula fluminea* inhabiting acidic waters (streams with pH 5.6) [55]. In a few extreme cases, shells were eroded to the extent that the mussels’ soft tissues were visible.

## Discussion

Our surveys documented the current distribution and change in historical range, densities, and preferred habitat of rare Texas species. Genetic analysis revealed that: (1) *F. lananensis* is not a valid species; (2) it is likely that only one *Fusconaia* species (*F. askewi*) is currently found in East Texas; (3) the presence of *F. flava* in East Texas is unlikely, however the species may still persist in the Red River basin and upper Trinity River; (4) *P. riddellii* was well-separated from *F. askewi* and instead grouped with the *P. sintoxia* clade.

### Genetic analysis

We found that the specimens from the Sabine and Neches drainages were genetically distinct from all other currently recognized *Fusconaia* species, as well as from the unusual sequences obtained by Burdick and White [17], and represented a distinct species. The relatively low percent difference from *F. burkei* reflects the shorter sequence for *F. burkei*, which consistently has a low difference from other sequences. Apart from it, all other *Fusconaia cox1* sequences differed from *F. askewi* and *F. lananensis* by more than 3.5 times as much as the largest difference within the *F. askewi-F. lananensis* group. In contrast, putative *F. askewi* sequences from the Calcasieu River in Louisiana matched closely sequences for *F. flava,* strongly suggesting that this population belongs in *F. flava* rather than *F. askewi*. The Calcasieu River runs between the Mississippi (specifically, the Red River) and the Sabine drainages, so faunal exchange could occur in either direction. Study of additional populations would be necessary to determine whether *F. askewi* is also present in the Calcasieu system or anywhere else east of the Sabine drainage.

All analyses strongly supported a group of *Fusconaia lananensis* and *F. askewi* (excluding the Calcasieu specimens). None of the analyses separated *F. askewi* from *F. lananensis*. Along with the low percentage difference (especially within the Neches drainage) and presence of morphologically intermediate specimens, this suggests that the *F. lananensis* is a subjective junior synonym of *F. askewi.* The distinguishing features noted by Frierson [36] would represent individual variation. Conversely, the specimens from the Calcasieu drainage are consistently strongly supported as closely related to *F. flava* and *F. cerina*. Current molecular data do not clearly distinguish between *F. cerina* and *F. flava*[17,47], so the Calcasieu population should probably be regarded as representing *F. flava*. The variations between Figures 1,2,3 show that relationships within *Fusconaia* are not well-resolved. Although the support is not strong, all analyses agree that *F. subrotunda* is basal, followed by a clade of *F. cor* and *F. cuneolus*. The remaining *Fusconaia* species, including *F. askewi* and *F. lananensis*, form a group with generally poorly resolved internal relationships. Thus, *F. askewi* and *F. lananensis* clearly belong in *Fusconaia*, are distinct from other currently recognized species (except each other), and are most closely related to the *F. cerina-F. flava* group, the *F. escambia-F. burkei* group, *F. masoni, F. ozarkensis*, and the unidentified *flava-*like *Fusconaia* from the Ozark region (*hebetata?*). Support for the genus *Fusconaia* is modest in the *cox1* only analysis (perhaps due to the partial sequences) but very high in the others. However, relationships of *Fusconaia* to other genera of Pleurobemini are poorly resolved, and the weakly supported relationships between genera are not consistent between analyses.

*Pleurobema riddellii* shows consistent but weakly supported affinity for members of the subgenus *Sintoxia-P. sintoxia, P. rubrum*, and *P. cordatum*. However, the *cox1* analysis shows that other specimens identified as *P. cordatum* are more distantly related to this group. This may reflect the difficulties of identifying species in the *P. cordatum* group. Ongoing genetic work on this group [56] shows further complications, but the morphological similarities of *P. riddellii* to the *P. cordatum* group [57] supports a relationship. Additionally, the only species of *Pleurobema* that occur in the lower Mississippi drainage are from the *P. cordatum* group [13], so the relationship also makes biogeographic sense.

At least four names older than *F. askewi* are available for *Fusconaia* species west of the Mississippi, besides *F. flava,* which was described from the Ohio drainage but occurs also in the upper Mississippi and west of it. *Fusconaia ozarkensis* (Call) is genetically and morphologically distinctive, but the remaining species have all been synonymized with or confused with *F. flava*: *Fusconaia fulgidus* (Lea), from the Red River at Alexandria, Louisiana; *F. hebetata* (Conrad), from Missouri (unfortunately, no information on which drainage); *F. chunii* (Lea), from the Trinity River at Dallas, Texas; and *F. friersoni* (Wright), from Bayou Pierre in the Red River system, De Soto Parish, Louisiana. Although the first three are generally regarded as synonyms of *F. flava*[16], as older names they would have priority over *F. askewi*; *F. friersoni* was published just before *F. askewi*, but appears to be a synonym of *P. riddellii* instead [49]. Burdick and White [17] sampled one population from the lower Red River drainage near Alexandria and found it genetically similar to *F. flava*. The present results for the Calcasieu system also suggest that *F. flava* occurs in the lower Red River system. Graf and Cummings [57] suggested that *F. hebetata* might be a valid species. Study of the populations in the Ozark region, building on the work of Utterback [58] and Graf [16], should determine whether the conchological variation in populations in this region can be correlated with the genetic divergence found by Burdick and White [17]. If so, *F. hebetata* and other names based on material from the Ozark region can be assigned to the appropriate population. However, as Burdick and White’s [17] sequences are quite distinct from those obtained in the present study for *F. askewi*, it seems safe to assume that *F. hebetata* is not applicable to the present material from Texas and Louisiana.

This leaves *F. chunii* as a possible senior synonym of *Fusconaia askewi* and *F. lananensis*. Howells *et al.*[14] synonymized *F. chunii* with *F. flava*, but Graf [16] identified their illustrated *F.* “*flava”* from Texas as different from true *F. flava*. We were unable to obtain live specimens from the Red River systems in Texas for genetic analyses. Specimens suggestive of *F. flava* from the Neches drainage, sampled in the present study, placed genetically with *F. askewi*. The Trinity system is immediately west of the Neches and the headwaters of the Sabine, and could easily have exchanged species through stream capture or other interaction. Stream capture occurs when a stream previously connected to one drainage system becomes connected to another, eventually becoming a part of the second drainage system [59]. However, the Trinity River headwaters also adjoin the Red River system in northern Texas. The lower Red River system in Louisiana has *F. flava*[17]. To the north of the Red River system is the Arkansas system, and the possible *F. hebetata* haplotype occurs in an Arkansas tributary. The picture is thus very complex, but it seems most likely that *F. chunii* is a senior synonym of *F. askewi*.

In contrast to the varying opinions on *Fusconaia* species, authors have generally agreed on recognizing *Pleurobema riddellii*. However, there has been some uncertainty about its affinities [13]. The present results provided moderate support for Frierson’s [60] suggestion that it is relatively closely related to the *Pleurobema cordatum* group. Most other work on this group has focused exclusively on the Mississippi drainage species and does not mention *P. riddellii*.

### Distribution, densities, size structure, and habitat

#### Fusconaia askewi

*F. askewi* is a regional endemic, historically known from the Sabine, Neches, Trinity and San Jacinto rivers in Texas [38] (Table 3, Figure 4A), and from Louisiana [13]. Simpson [50] lists *F. askewi* range from western Louisiana to eastern Texas with type locality as Village Creek, Hardin Co., and the Sabine River, Texas. Strecker [34] recorded this species in the Angelina, Sabine and Navasota rivers, and from Kickapoo Creek. Neck [49] reported *F. askewi* as locally common, but noted that the status over its entire range was unclear. During our surveys we found live *F. askewi* in four drainages in eastern and northeastern Texas (Table 3, Figure 4B). This species was locally abundant, often dominated mussel assemblages, and several populations were reproducing. The most typical substrate for the species was sand, sand and silt, and gravel with sand.

*Fusconaia lananensis* was described by Frierson in 1901 [36], after the first account of Texas unionids was published [37]. Frierson collected 200 specimens of *F. lananensis* from Lanana and Bonita creeks near Nacogdoches, Texas [36]. Strecker [34] found live *F. lananensis* in Lanana Creek, and in the San Jacinto River. In 1990s, few live mussels were found in Attoyac Bayou and Sandy Creek (Angelina River drainage) [51], and 36 live mussels were found in Village Creek [15]. We found live mussels that fit the description of “*F. lananensis*” in several waterbodies in East Texas. Due to the similar shell morphologies of *F. askewi* and *F. lananensis*, field identification between the two nominal species was very challenging, which is not surprising considering their genetic similarity. Frierson [36] reports that “*Q[uadrula] lananensis* is closely allied to *Q*. *askewi* Marsh, both by its conchological and anatomical characteristics. It may be differentiated from that shell by being longer, more compressed, more oblique, and its shell is never so inflated and thickened in front as *askewi* and not so acutely angled on the posterior ridge. Internally, *lananensis* is rose-colored nearly invariably and the color is uniformly spread over its surface. *Askewi* is mostly white, and, when colored (pink) the color is almost always confined exterior to the pallial line. Finally, *Q*. *askewi* never possess those peculiar pearly excrescences, which seem to belong to *lananensis*”. We observed several patterns in nacre coloration of *Fusconaia* from East Texas drainages. There were three forms recorded in the Neches drainage: with entirely white nacre, solid rose/pink, and the form with the pink extrapallial ring described by Frierson [36]. Practically the entire *Fusconaia* population in the Sabine River had white nacre, while almost none of the Trinity *Fusconaia* showed the pink extrapallial ring (most of them were white, and a few - solid pink). Therefore, we saw the same features (e.g., pearly excrescences and rose-colored nacre) in both species, with many intermediate forms that were impossible to separate, suggesting that *F. lananensis* may not be a valid species. This suggestion was supported by our genetic analysis. Habitat and substrate preferences of both *Fusconaia* spp. were found to be similar as well.

#### Pleurobema riddellii

This species is a regional endemic, found in Texas and Louisiana [14,51]. Singley [37] recorded *P. riddellii* in Village Creek only; Strecker recorded the species from the Angelina, Sabine, San Jacinto and Trinity rivers in East Texas [34] (Figure 6A). NatureServe reports a substantial recent decline in this species [61]. During our surveys, we found a total of 132 live *P. riddellii* in one East Texas river basin (the Neches River), but not at the sites we surveyed on the Trinity River (Figure 6B). *Pleurobema riddellii* has probably been extirpated from the San Jacinto River. This species was not locally abundant, and not dominant in mussel assemblages. Although most populations were comprised of older animals, several populations were reproducing. *Pleurobema riddellii* was found exclusively in lotic waters, in relatively shallow areas, most often in sand, or in a mixture of sand, gravel and silt.

### Conservation priorities

#### Fusconaia askewi

The American Fisheries Society considers *F. askewi* and *F. lananensis* to be of special concern [4], and both species are currently listed as state threatened [8] and as near-threatened by the IUCN [62]. Our recent surveys classified these species as rare (species that were found at low densities in 1 to 9 Texas waterbodies) based on their occurrence and density [5]. The U.S. Fish and Wildlife Service found that substantial scientific information was presented indicating that listing of *F. lananensis* may be warranted due to the present or threatened destruction, modification, or curtailment of its habitat or range [10], and a status review for the species was initiated in 2009. However, our study suggested that *F. lananensis* is not a valid species and it is likely that only one *Fusconaia* species (*F. askewi*, senior synonym *F. chunii*) is currently present in East Texas, thus simplifying conservation efforts. Although we found that *F. askewi* still inhabits four river basins in eastern and northeastern Texas and can be locally abundant, its distribution range has been reduced in the last 80 years: the species have been extirpated from a number of waterbodies in Texas, including Lanana and Bonita creeks, the San Jacinto and Navasota rivers, and Kickapoo Creek (Figure 4). The distribution of *F. askewi* in the Trinity River has been also reduced in the last 40 years (Figure 4). The species has been extirpated from much of its former range in the upper Trinity River north of SR-7 (Leon/Houston Counties), and appears to be completely absent from the river south of Lake Livingston (D. Barclay, personal observations).

#### Pleurobema riddellii

This species was found in only one East Texas drainage (the Neches River), and at very low densities. During the last 80 years the distribution range of *P. riddellii* has been dramatically reduced, and this species has been extirpated from several East Texas waterbodies where it occurred historically (Figure 6). Notably, some of these waterbodies (e.g., San Jacinto River) that lost both *F. askewi* and *P. riddellii*, are the most highly populated in Texas [19]. At the beginning of 20^th^ century, the San Jacinto River was a home for 29 unionid species, but due to extensive mining, deforestation, damming and urbanization, it lost almost 70% of its former unionid diversity [19]. The U.S. Fish and Wildlife Service found that listing of *P. riddellii* as threatened or endangered may be warranted due to the present or threatened destruction, modification, or curtailment of its habitat or range resulting from general human modification of the water and adjacent land, siltation, impoundments, and water pollution [9,10], however it is currently listed as threatened only at the state level [8].

Currently East Texas has predominantly forested watersheds with little urbanization, both factors being important for maintaining the health of aquatic environments [63]. Not surprisingly, this part of Texas is the hotspot for the state’s unionid diversity where almost every river supports from 17 to 28 species [19]. However, Texas is one of the fastest growing states in the nation. The urban population in Texas nearly doubled in the last 30 years [64], with a 21% increase in urbanization since 1990 [65]. Along with growing urbanization, it is predicted that > 20 million ha of U.S. forest will be developed over the next 50 years [66,67], and > 11% of private forests, mostly in the South, could experience substantial increases in housing density by 2030 [68,69]. Considering growing development and water demand, the best measure for conservation of both *F. askewi* and *P. riddellii* would be by controlling deforestation, urbanization and water diversion in East Texas watersheds, and particularly the Neches River.

## Competing interests

The authors declare that they have no competing interests.

## Authors’ contributions

LEB and AYK designed the study and surveyed sites state-wide. DB surveyed additional sites in East Texas. DC carried out the molecular genetic studies and their interpretation. LEB, AYK and DC led, and DB edited the writing. All authors read and approved the final manuscript.

## Supplementary Material

Additional file 1 **Sequences used for genetic analysis [**[42,47,48,56,70-78]**].** Click here for file
