# Supplementary material for: Distribution, genetic analysis and conservation priorities for rare Texas freshwater molluscs in the genera Fusconaia and Pleurobema (Bivalvia: Unionidae)
Source: Aquat Biosyst. 2012 Jun 25;8:12. doi: 10.1186/2046-9063-8-12 (PMC3422191; doi:10.1186/2046-9063-8-12)
Supplement: Additional file 1 — Sequences used for genetic analysis [[42,47,48,56,70-78]]. [file 2046-9063-8-12-S1.doc]

**Additional file 1 -** **Sequences used for genetic analysis.**

| **Taxon** | **Source** | **Accession** | **Gene** |
| --- | --- | --- | --- |
| ***Amblema elliottii* (Lea)** | [70] | AY655086 | *nad1* |
| ***Amblema elliottii*** | [70] | AY654991 | *cox1* |
| ***Amblema plicata* (Say)** | [42] | AY158796 | *nad1* |
| ***Amblema plicata*** | [71] | EF033258 | *cox1* |
| ***Cyrtonaias tampicoensis* (Lea)** | [70] | AY655090 | *nad1* |
| ***Cyrtonaias tampicoensis*** | [71] | EF033259 | *cox1* |
| ***Elliptio (Eurynia) dilatata* (Rafinesque)** | [72] | AF156506 | *cox1* |
| ***Elliptio (Eurynia) dilatata*** | [73] | DQ385872 | *nad1* |
| ***Elliptio arca* (Conrad)** | [70] | AY655093 | *nad1* |
| ***Elliptio arca*** | [70] | AY654995 | *cox1* |
| ***Elliptio crassidens* (Lamarck)** | [74] | EU377568 | *cox1* |
| ***Elliptio crassidens*** | UAM3527 | *JN180972 | *nad1* |
| ***Fusconaia "askewi"* (Marsh) 3391** | UAM3391 | JN192389 | *nad1* |
| ***Fusconaia “askewi”* 3392** | [47] | HM230367 | *cox1* |
| ***Fusconaia “askewi”* 3392** | [47] | HM230411 | *nad1* |
| ***Fusconaia “askewi”* 3395** | [47] | HM230366 | *cox1* |
| ***Fusconaia askewi* Sab1** |  | *JN180975 | *nad1* |
| ***Fusconaia askewi* Sab1 2** |  | *JN180994, *JN180995 | *cox1* |
| ***Fusconaia askewi* Sab2** |  | *JN180976 | *nad1* |
| ***Fusconaia askewi* Sab3** |  | *JN180996 | *cox1* |
| ***Fusconaia askewi* Sab4** |  | *JN180997 | *cox1* |
| ***Fusconaia askewi* Sab5** |  | *JN180998 | *cox1* |
| ***Fusconaia askewi* Sab5** |  | *JN180977 | *nad1* |
| ***Fusconaia askewi* TS131 133** |  | *JN180989, JN180991 | *cox1* |
| ***Fusconaia askewi* TS166** |  | *JN180992 | *cox1* |
| ***Fusconaia askewi* TS219** |  | *JN180974 | *nad1* |
| ***Fusconaia askewi* TS233** |  | *JN180973 | *nad1* |
| ***Fusconaia askewi* TS233 TS130 TS204** |  | *JN180986, *JN180993, *JN180988 | *cox1* |
| ***Fusconaia burkei* (Walker)** | [75] | AF232802 | *cox1* |
| ***Fusconaia burkei*** | [42] | AY158793 | *nad1* |
| ***Fusconaia cerina* (Conrad)** | [70] | AY613823 | *cox1* |
| ***Fusconaia cerina*** | [70] | AY613792 | *nad1* |
| ***Fusconaia cerina* LA** | [47] | HM230368 | *cox1* |
| ***Fusconaia cor* (Conrad)** | [70] | AY655096 | *nad1* |
| ***Fusconaia cor*** | [70] | AY654997 | *cox1* |
| ***Fusconaia cor 2*** | [47] | HM230369 | *cox1* |
| ***Fusconaia cuneolus* (Lea)** | [70] | AY655097 | *nad1* |
| ***Fusconaia cuneolus*** | [70] | AY654998 | *cox1* |
| ***Fusconaia escambia* Clench & Turner** | [47] | HM230413 | *nad1* |
| ***Fusconaia Escambia*** | [75] | AF232816 | *cox1* |
| ***Fusconaia flava* (Rafinesque)** | [47] | HM230414 | *nad1* |
| ***Fusconaia flava* 1** | [72] | AF156510 | *cox1* |
| ***Fusconaia flava* H1681** | [71] | EF033261 | *cox1* |
| ***Fusconaia flava* MO** | [47] | HM230370 | *cox1* |
| ***Fusconaia hebetata?* (Conrad) *Ff8*** | [17] | DQ298531 | *cox1* |
| ***Fusconaia hebetata? Ff9*** | [17] | DQ298532 | *cox1* |
| ***Fusconaia lananensis* (Frierson) TS129 132 179 203** |  | *JN180987, *JN180990, *JN180984, *JN180985 | *cox1* |
| ***Fusconaia lananensis* TS129 TS179** |  | *JN180980, *JN180981 | *nad1* |
| ***Fusconaia lananensis* TS203** |  | *JN180982 | *nad1* |
| ***Fusconaia masoni* (Conrad)** | [47] | HM230415 | *nad1* |
| ***Fusconaia masoni*** | [51] | EF619921 | *cox1* |
| ***Fusconaia ozarkensis* (Call)** | [47] | HM230416 | *nad1* |
| ***Fusconaia ozarkensis*** | [47] | HM230373 | *cox1* |
| ***Fusconaia ozarkensis 2*** | [47] | HM230372 | *cox1* |
| ***Fusconaia subrotunda* (Lea)** | [70] | AY613794 | *nad1* |
| ***Fusconaia subrotunda*** | [70] | AY613824 | *cox1* |
| ***Fusconaia subrotunda* Pal** | Allegheny River, PA | *JN181001 | *cox1* |
| ***Fusconaia subrotunda* Pal** | Allegheny River, PA | *JN180978 | *nad1* |
| ***Fusconaia subrotunda* Pas** | Allegheny River, PA | *JN181002 | *cox1* |
| ***Fusconaia subrotunda* Pas** | Allegheny River, PA | *JN180979 | *nad1* |
| ***Hemistena lata* (Rafinesque)** | [70] | AY613796 | *nad1* |
| ***Hemistena lata*** | [70] | AY613825 | *cox1* |
| ***Lampsilis ovata* (Say)** | [76] | AF385111 | *cox1* |
| ***Lampsilis ovata*** | [70] | AY613797 | *nad1* |
| ***Plectomerus dombeyanus* (Valenciennes)** | [70] | AY655110 | *nad1* |
| ***Plectomerus dombeyanus*** | [71] | EF033252 | *cox1* |
| ***Plethobasus cyphyus* (Rafinesque)** | [70] | AY613799 | *nad1* |
| ***Plethobasus cyphyus*** | [70] | AY613828 | *cox1* |
| ***Pleurobema beadleianum* (Lea)** | [5] | DQ383429 | *cox1* |
| ***Pleurobema beadleianum*** | Red Creek, Washington Co. AL | *JN180983 | *nad1* |
| ***Pleurobema clava* (Lamarck)** | [70] | AY613802 | *nad1* |
| ***Pleurobema clava*** | [48] | AF231754 | *cox1* |
| ***Pleurobema decisum* (Lea)** | [73] | DQ383466 | *nad1* |
| ***Pleurobema decisum*** | [70] | AY613832 | *cox1* |
| ***Pleurobema furvum* (Conrad)** | [70] | AY613833 | *cox1* |
| ***Pleurobema furvum*** | [70] | AY613806 | *nad1* |
| ***Pleurobema georgianum* (Lea)** | [70] | AY613834 | *cox1* |
| ***Pleurobema georgianum*** | [70] | AY613807 | *nad1* |
| ***Pleurobema hanleyianum* (Lea)** | [70] | AY613836 | *cox1* |
| ***Pleurobema hanleyianum*** | [70] | AY613809 | *nad1* |
| ***Pleurobema oviforme* (Conrad)** | [70] | AY655017 | *cox1* |
| ***Pleurobema oviforme*** | [70] | AY613810 | *nad1* |
| ***Pleurobema perovatum* (Conrad)** | [77] | JN180999 | *cox1* |
| ***Pleurobema perovatum*** | [70] | AY613811 | *nad1* |
| ***Pleurobema pyriforme* (Lea)** | [73] | DQ383468 | *nad1* |
| ***Pleurobema pyriforme*** | [73] | DQ383468 | *cox1* |
| ***Pleurobema rubellum* (Conrad)** | [70] | AY613840 | *cox1* |
| ***Pleurobema rubellum*** | [70] | AY613813 | *nad1* |
| ***Pleurobema strodeanum* (Wright)** | [70] | AY613843 | *cox1* |
| ***Pleurobema strodeanum*** | [70] | AY613817 | *nad1* |
| ***Pleurobema taitianum* (Lea)** | [70] | AY613844 | *cox1* |
| ***Pleurobema taitianum*** | [70] | AY613818 | *nad1* |
| ***"Pleurobema” collina* (Conrad)** | [78] | EU414270 | *nad1* |
| ***"Pleurobema” collina*** | [70] | AY613830 | *cox1* |
| ***"Pleurobema” stabile* (Lea)** | [73] | AY613816 | *nad1* |
| ***"Pleurobema” stabile*** | [73] | AY613842 | *cox1* |
| ***Pleuronaia barnesiana* (Lea)** | [70] | AY613791 | *nad1* |
| ***Pleuronaia barnesiana*** | [47] | HM230418 | *nad1* |
| ***Pleuronaia dolabelloides* (Lea)** | [70] | AY655051 | *nad1* |
| ***Pleuronaia dolabelloides*** | [70] | AY613827 | *cox1* |
| ***Pleuronaia gibbera* (Lea)** | [70] | AY613808 | *nad1* |
| ***Pleuronaia gibbera*** | [5] | DQ383432 | *cox1* |
| ***Popenaias popeii* (Lea)** | [70] | AY655118 | *nad1* |
| ***Popenaias popeii*** | [70] | AY655020 | *cox1* |
| ***Pleurobema (Sintoxia) cordatum* (Rafinesque) 2926** | [51] | EF619917 | *cox1* |
| ***Pleurobema (Sintoxia) cordatum* 2927** | [51] | EF619918 | *cox1* |
| ***Pleurobema (Sintoxia) cordatum*** | [70] | AY613804 | *nad1* |
| ***Pleurobema (Sintoxia) cordatum*** | [70] | AY613831 | *cox1* |
| ***Pleurobema (Sintoxia) plenum* (Lea) 01** | [51] | EF619920 | *cox1* |
| ***Pleurobema (Sintoxia) plenum* 03** | [51] | EF619919 | *cox1* |
| ***Pleurobema (Sintoxia) riddellii* (Lea) TS186** | [77] | JF326434 | *cox1* |
| ***Pleurobema (Sintoxia) riddellii* TS187** | Village Creek, Hardin Co., TX | *JN181000 | *cox1* |
| ***Pleurobema (Sintoxia) riddellii* TS186** | [13] | JF326444 | *nad1* |
| ***Pleurobema (Sintoxia) rubrum* (Rafinesque)** | [70] | AY613841 | *cox1* |
| ***Pleurobema (Sintoxia) rubrum* 1** | [77] | JF326445 | *nad1* |
| ***Pleurobema (Sintoxia) rubrum* 2** | [70] | AY613814 | *nad1* |
| ***Pleurobema (Sintoxia) rubrum* 3** | [70] | AY655117 | *nad1* |
| ***Pleurobema (Sintoxia) sintoxia* (Rafinesque) H1766** | [73] | EF033253 | *cox1* |
| ***Pleurobema (Sintoxia) sintoxia* 1** | [72] | AF156508 | *cox1* |
| ***Pleurobema (Sintoxia) sintoxia* 2** | [72] | AF156509 | *cox1* |
| ***Pleurobema (Sintoxia) sintoxia*** | [70] | AY613815 | *nad1* |
